# Description of the combined evidence-based, theory-based and person-based approaches used to develop a behavioural intervention package to support non-allergist healthcare workers to remove incorrect penicillin allergy records from medical and surgical adult inpatients in a UK hospital

**DOI:** 10.1136/bmjopen-2024-096452

**Published:** 2025-07-30

**Authors:** Neil Powell, Mathew Upton, Bridie Kent, Jonathan Sandoe, Sarah Tonkin-Crine

**Affiliations:** 1 Department of Pharmacy, Royal Cornwall Hospital, Truro, UK; 2 University of Plymouth, Plymouth, UK; 3 Nursing and Midwifery, Plymouth University, Plymouth, UK; 4 Department of Microbiology, University of Leeds Faculty of Medicine and Health, Leeds, UK; 5 Nuffield Department of Primary Care Health Science, University of Oxford, Oxford, UK

**Keywords:** Implementation Science, INFECTIOUS DISEASES, Pharmacists

## Abstract

**Abstract:**

**Objectives:**

To develop a behavioural intervention package to support non-allergist healthcare workers (HCWs) to remove incorrect penA records from medical and surgical adult inpatients. This paper describes the development of the penicillin allergy de-labelling (PADL) intervention and the implementation intervention that will support non-allergist-delivered PADL.

**Design:**

We combined evidence-based, theory-based and person-based approaches. Qualitative research with healthcare professionals and patients explored barriers and enablers to implementation of the proposed PADL pathway. Key intervention design objectives and the key features of the implementation intervention required to achieve each objective were then developed and captured as guiding principles. We produced a logic model, integrating the theoretical domains framework to identify the behavioural influences on PADL and the behaviour change wheel to show how the implementation intervention is hypothesised to address the target behaviours. The implementation intervention package was then reviewed by stakeholders and topic experts for further refinement and optimisation. Finally, we outline how the implementation intervention will be evaluated.

**Setting:**

Single-centre District General Hospital in the SW England servicing a rural community of 575 000 people without local allergy services.

**Results:**

HCWs reported PADL needed to be structured, standardised, evidence based and supported by hospital approved guidelines with easy to access patient information leaflets, supported by a sustained programme of education and training with named PADL leaders and visible PADL champions. Patients wanted a good explanation of the benefits and risks of testing and the benefits of having their ‘penA’ record removed. The identified HCW target behaviours were: taking a penA allergy focused history and to risk assess the patient’s penA history; to then either de-label the patient on history alone (direct de-label; DDL) or prescribe a direct oral challenge (DOC) dose; to perform baseline and post-test observations and counsel the patient on the risks of penA records and on the risks and the benefits of PADL. We identified barriers to target behaviours that we considered both important and modifiable, which included: lack of confidence in taking a penA focused history, PADL not viewed as a priority, low confidence with differentiating low-risk and high-risk penA histories, concerns about the safety of DOC, a requirement for senior support for nurses to deliver the observations and senior support for the other HCWs to deliver PADL, access to an expert for advice when required, a lack of PADL champions to promote PADL, and PADL not being supported by the organisation. The identified patient target behaviours were acceptance of the opportunity to be de-labelled via either DDL or DOC and willingness to take penicillin when prescribed. We developed intervention components to target the HCW and patient target behaviours which included: Education, expert advice made available from Infection specialists, a named PADL champion, hospital endorsed PADL guideline with necessary tools to enable PADL and patient information leaflets. The implementation intervention was further optimised through workshops with PADL researchers and stakeholders. The Consolidated Framework for Implementation Research outcome addendum was used to define both implementation intervention and PADL intervention outcomes.

**Conclusions:**

We have developed a theory-based and stakeholder-developed implementation intervention to support inpatient PADL delivered by a multiprofession workforce. The intervention will be tested in a single hospital and scalability explored.

**STRENGTHS AND LIMITATIONS OF THIS STUDY**

- We followed a systematic process to development of the intervention using the views of local patients and local healthcare workers and behavioural theory.
- We engaged large and diverse stakeholder groups to refine the intervention which included educationalists, topic experts with practical and research experience with delivering penicillin allergy de-labelling and predominantly senior clinicians.
- There were few pharmacists, Medicines Optimisation Pharmacy Technicians and nurse stakeholders in the refinement stages, but we feel we had reasonable representation from these groups in the predevelopment stages in the qualitative studies.
- We had patient involvement in refining the patient information leaflets only and not the implementation intervention. But again, we had good patient representation in the predevelopment qualitative study.

## Introduction

 Penicillin allergy (penA) is common, with 15% of hospitalised patients reporting penA, but the majority, 95%, are able to take penicillin after formal penA testing.^1^,^3^ Having a penA recorded is associated with patient, health system and wider societal harms which include the use of more broad-spectrum antibiotics, increased length of hospitalisation, higher rates of admission to intensive care units and hospital readmission, multidrug-resistant or opportunistic infection and increased mortality, with patients incurring significantly higher drug or hospital-related costs.^4^ Penicillin antibiotics are often first-line treatment for many common infections because penicillin antibiotics are effective, well-tolerated and inexpensive. The associated harms of penA records are thought to be due to avoidance of penicillin antibiotics in favour of alternative antibiotics which are often broader spectrum and with a higher potential for side effects and to drive antimicrobial resistance.^5 6^ Due to the large number of patients with penA records, the fact that most are incorrect, and their negative impact on antimicrobial stewardship (AMS) and patient care, the removal of incorrect penA records, also called penicillin allergy de-labelling (PADL), is an AMS priority in the UK and globally.^7 8^

Traditionally, PADL has been the role of the allergists and involved skin testing prior to drug provocation testing, but the scale of the problem, the resource-intensive nature of traditional penA testing and the paucity of allergists in the UK and elsewhere makes de-labelling at scale using this approach impractical.^9 10^ In 2008, direct oral challenge (DOC) testing without prior blood tests and skin testing was shown to be safe and less resource intense than traditional penA testing in patients with a low-risk penA history which meant PADL could be delivered to more patients and potentially by non-allergist healthcare workers (HCWs).^11^ A systematic review published in 2023 reported on the safety of non-allergist-delivered PADL for 713 patients on history alone and 1288 via DOC.^12^ Non-allergist-delivered PADL is now well supported with several published national and international consensus guidelines and toolkits that facilitate and support the delivery of PADL by non-allergists.^13^,^18^

Supporting HCWs to deliver PADL requires a complex intervention to target a number of both HCW and patient behaviours and it requires upskilling of the multidisciplinary healthcare workforce to deliver PADL.^19^ To be successfully implemented, as is true of other complex interventions, requires a theoretical deconstruction of intervention components and an exploration of how these components interact with the intended context.^19^ We aimed to develop a behavioural intervention package for a District General Hospital in England that would enable and support non-allergist HCWs to remove incorrect penA records from medical and surgical adult inpatients, enabling the prescription of penicillin when it is first line therapy. This paper describes the development of the PADL intervention and the implementation intervention that will support non-allergist-delivered PADL.^20^

## Methods and results

We followed an integrated approach to the development of this implementation intervention that combined theory-based, evidence-based and person-based approaches (PBAs).^21 22^ We were specifically guided by techniques used in the PBA. This approach has been successfully used to develop other behavioural AMS interventions, including a PADL intervention.^23^,^25^ We used the theoretical domains framework (TDF) and the behaviour change wheel (BCW) to help identify the behavioural influences on PADL.^26 27^ The TDF is a theory-informed approach to identify determinants of behaviour.^26^ The BCW provides a systematic way of characterising interventions, their mechanisms of action and link intervention components to outcomes.^27^ The guidance for reporting intervention development studies in health research (GUIDED) was used for reporting.^28^

We undertook four stages, applying the PBA, in the development of the implementation intervention. Stage 1 involved a systematic review of the literature to identify non-allergist-delivered PADL patient pathways and toolkits, and also undertook qualitative research with healthcare professionals and patients in the study hospital to explore barriers and enablers to implementation of a proposed intervention and to identify influences on the target behaviour(s).^22^

In stage 2, the key intervention design objectives and the key features were captured as guiding principles. The components of the implementation intervention required to achieve each objective were then developed. We produced a logic model, integrating behavioural theory to show how the implementation intervention is hypothesised to address the target behaviours.

In stage 3, the implementation intervention package was reviewed by both stakeholders and topic experts to seek feedback on further refinement and optimisation of the implementation intervention to make it more attractive, persuasive and feasible to implement. Finally, in stage 4, we outline how the implementation intervention will be evaluated. Table 1 summarises the intervention and the implementation intervention development steps and planned evaluation.^22^

**Table 1:** The implementation intervention development steps^22^

Stages	Steps	Person based approach	Theory/ framework
Collating evidence	Defining the penicillin allergy de-label (PADL) pathway for inpatients. Using the Effective Practice and Organisation of Care (EPOC) taxonomy of health systems interventions to catalogue interventions used to support PADL intervention. Identifying influences on HCW and patient behaviours related to delivery of PADL.	Intervention Planning: Systematic review of non-allergist-delivered PADL. Review of PADL patient pathways in the literature. Qualitative research with target users.	
Intervention planning, design and development	Defining the PADL components in behavioural terms, identifying target users and behaviours, and the influences on those behaviours. Creating guiding principles and theoretical modelling (logic model). Identifying intervention components. Developing implementation intervention materials to support the target PADL behaviours.	Intervention design: Identify target users and behaviours. Formulating guiding principles Behavioural analysis and construction of logic model Develop implementation intervention materials.	TDF and BCW
Intervention optimisation	Refine the implementation intervention through expert (implementation science experts, educationalists, researchers with expertise in PADL research) and stakeholder (local patients and healthcare workers) workshops.	Intervention optimisation: Expert and stakeholder workshops to refine materials and implementation plan.	
Implementing and evaluating the implementation intervention	Implementing the implementation intervention in real-life context. Mixed-methods evaluation.	Mixed methods process evaluation: Qualitative research Quantitative research	CFIR

BCW, behaviour change wheel; CFIR, Consolidated Framework for Implementation Research outcome addendum; HCW, healthcare worker; PADL, penicillin allergy de-labelling; TDF, theoretical domains framework.

### Patient and public involvement

During the funding application process patients with penA records who had had inpatient hospital stays were consulted in focus groups to discuss their experiences of having a penA record and their acceptance of the proposed testing methods in hospital, and the approaches that might be taken by HCWs. Patient focus groups were convened to discuss the findings of completed work packages completed prior to this stage. Patients with penA records who were offered testing during a pilot study were invited to share their experiences in the qualitative interviews during which patient priorities, experiences and preferences for penA testing were explored and used to optimise the implementation intervention described in this manuscript. Patients were partners in developing the patient information leaflets used as part of the implementation intervention.

### Stage 1: collating evidence

#### Methods

##### Systematic review

We systematically reviewed the literature to determine the effectiveness and safety of non-allergist HCW delivery of PADL.^12^ We found PADL by non-allergists to be efficacious and safe but found testing strategy heterogeneity between the studies.^29^ We adopted the Scottish PADL toolkit and testing protocol, made local modifications to the testing protocol and the toolkit, and piloted PADL in the study hospital.^30^ In the systematic review, we synthesised the reported interventions that facilitated PADL, for example, HCW training, and used those to inform the intervention and implementation intervention.^12^

##### Qualitative methods

Two qualitative studies were undertaken, led by NP; 1 exploring barriers and enablers to PADL with 19 patients who had recently been offered non-allergist ward-based PADL and 1 with 23 HCWs exploring barriers and enablers to delivering PADL.^31 32^ The aim was to explore the experiences of those patients and to explore the perspectives of HCWs across medical specialties concerning managing patients with penA records and delivering PADL.^31 32^

### Results

#### Main findings from HCW interviews

The behaviours required to deliver PADL aligned with HCW roles and with inpatient pathways. Senior doctors reported feeling more confident to deliver PADL compared with more junior doctors and pharmacists. HCWs reported that PADL needed to be structured, standardised, evidence-based and preferably with a validated decision support tool. They said PADL needs to be supported by hospital approved guidelines, easy to access patient information leaflets to aid patient counselling and needs promoting as safe and in patients’ best interest. PADL needs to be supported by a sustained programme of education and training via multiple channels, reminders and prompts (ward pharmacists, electronic prescribing and medicines administration (EPMA) system, patients), with visible, named PADL leaders and visible PADL champion(s). Raising public awareness about the benefits of PADL may empower patients to ask HCWs about their penA. Good communication of a penA test result with the patient’s general practitioner (GP) was seen as important. If time is limited, especially for more senior clinicians, then brief questions to triage patients before referring for testing were seen to be helpful, perhaps to a dedicated PADL team. If nursing time was limited, then using nursing and medical students to do the post DOC monitoring may facilitate PADL.

#### Main findings from patient interviews

Most were unaware of the negative impact of penA on antibiotic use and had not had negative experiences themselves. Patients did express a desire to receive the best treatment, and so patients wanted a good explanation of the benefits and risks of testing and the benefits of having their ‘penA’ record removed, both verbally and in writing. Some patients said that they had declined testing either due to their advanced age, their multiple comorbidities or high acuity illness and that nothing would change their minds, expressing concerns that if they were to have a reaction, they feared that they would find it difficult to recover. A belief that testing and receiving penicillin would be beneficial motivated some patients to undergo PADL. Those who had agreed to PADL reported they had confidence in the PADL process and confidence in their negative PADL test result and were grateful for the increased treatment options available to them.

### Stage 2: implementation intervention planning, design and development

#### Defining the target users, target behaviours and influences on behaviours

##### Methods

**Figure 1:**
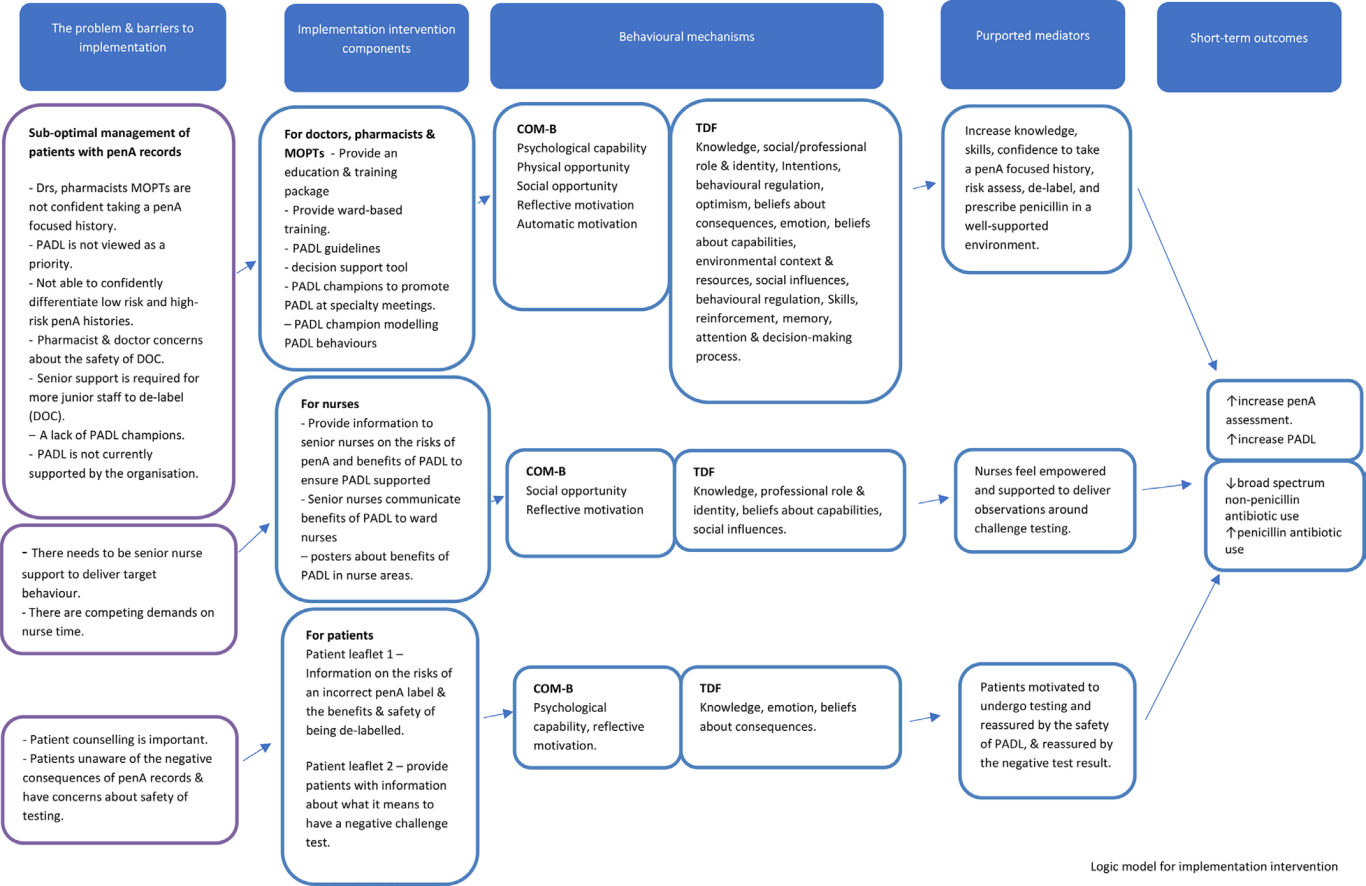
Penicillin allergy de-labelling (PADL) implementation intervention logic model summarising the implementation intervention components, behavioural mechanisms, purported mediators for change and outcomes. MOPTs, Medicines Optimisation Pharmacy Technicians; TDF, theoretical domains framework.

**Figure 2:**
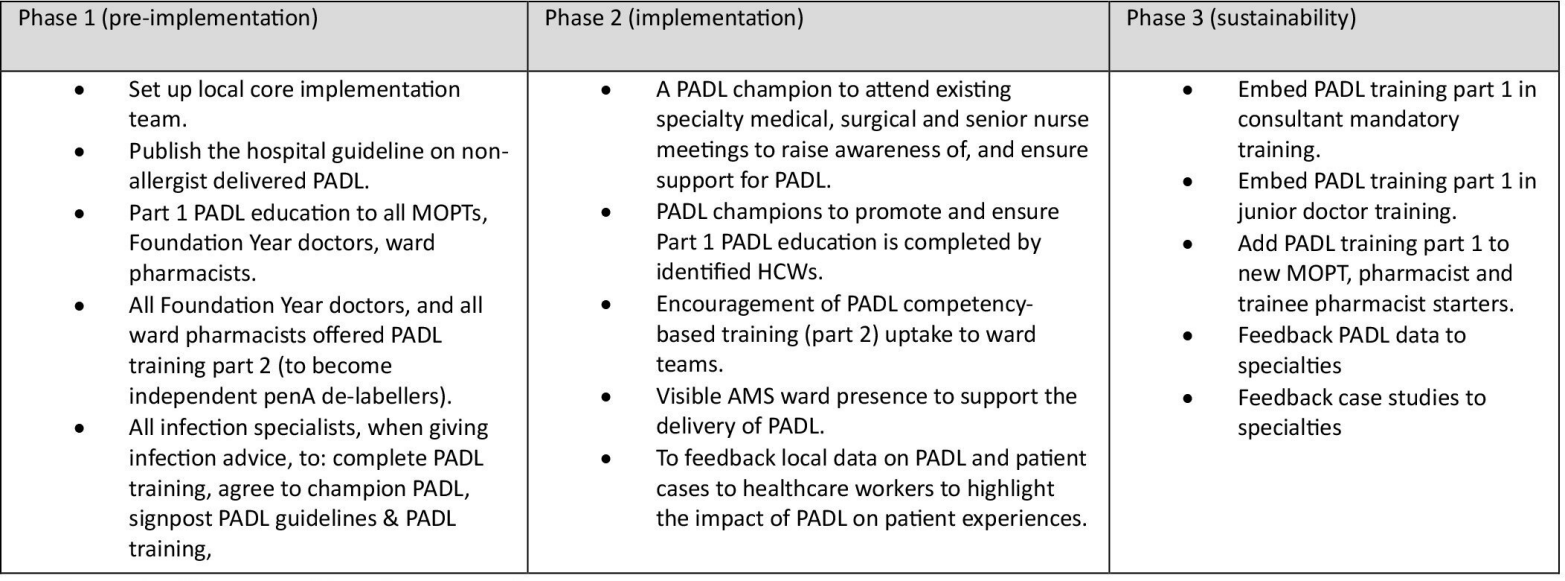
Phases of implementation. AMS, antimicrobial stewardship; HCWs, healthcare workers; MOPTs, Medicines Optimisation Pharmacy Technicians; PADL, penicillin allergy de-labelling.

The core intervention development research team included a consultant medical microbiologist and a health psychologist, neither working at the study hospital, and a consultant antimicrobial pharmacist working at the study hospital. We used the evidence collated in the qualitative studies and the expertise of the research team in the initial planning of the intervention. We undertook a behavioural analysis to define the target behaviours, identify which HCWs would enact the target behaviours (target users), also including patient behaviours, and then defined the intervention components that might influence the target behaviours. We identified the intervention function responsible for the action (‘intervention ingredient’) and mapped those to COM-B and the TDF.^26 27^ From this, we developed a logic model to provide an overview of how the implementation intervention is hypothesised to enable the delivery of PADL (figure 1). Finally, we drafted a phase of implementation plan to guide deployment of the intervention (figure 2).

##### Results

###### Target behaviours, HCWs

The identified target behaviours were to take a penA allergy focused history and to risk assess the patient’s penA history; to then either de-label the patient on history alone or prescribe a DOC dose; to perform baseline and post-test observations and counsel the patient on the risks of penA records and on the risks and the benefits of PADL. We identified barriers to target behaviours that we considered both important and modifiable. These included lack of confidence taking a penA focused history, PADL not viewed as a priority, low confidence with differentiating low-risk and high-risk penA histories, concerns about the safety of DOC, a requirement for senior support for nurses to deliver the observations and senior support for the other HCWs to deliver PADL, access to an expert for advice when required, a lack of PADL champions to promote PADL and PADL not being supported by the organisation (see behavioural analysis; online supplemental appendix table A1).

###### Target users

The qualitative interviews explored the PADL pathway and the roles of HCWs in undertaking the behaviours required to deliver PADL. Taking a penA focused history was seen to align with Medicines Optimisation Pharmacy Technicians (MOPTs), pharmacists and doctors’ roles. Risk assessing penA histories, de-labelling, counselling patients, updating penA status and communicating new penA status to primary care was seen to align with doctors and pharmacists’ roles. Undertaking the prechallenge and postchallenge observations was seen to align with nurse roles.

###### Target behaviours, patients

The identified target behaviours (see behavioural analysis; online supplemental appendix table A2) were acceptance of the opportunity to be de-labelled via either DDL (direct de-label) or DOC and willingness to take penicillin when prescribed. Concerns about the safety of testing, particularly among patients with advanced age, comorbidities or high acuity illness and a lack of awareness of the negative impact of taking alternatives to penicillin made some patients reluctant to agree to PADL.

###### Intervention components to target HCW behaviours

Education, provided in two parts: 25 min, prerecorded, training module plus a 40 min face-to-face case study-based competency-based training. Presentations on the benefits and the risks of PADL will be delivered to clinical nurse, medical and pharmacy leadership and to medical specialty groups to ensure senior and specialty level support for PADL. Expert advice will be made available from infection specialists (antimicrobial pharmacists, medical microbiologists and infectious diseases consultant) and the consultant antimicrobial pharmacist will be the named PADL lead and champion PADL, supported by the other infection specialists. There will be a hospital endorsed PADL guideline published containing all necessary tools to enable PADL (eg, risk assessment tool). Patient information leaflets, with key counselling points, will be produced and made available in the hospital PADL guidelines to facilitate verbal and written counselling of patients on the risks and benefits of PADL (see table 3).

###### Guiding principles

Using the PBA, we identified ‘guiding principles’ (table 2) to encapsulate the most important insights from the behavioural analysis to communicate the key objectives and distinctive features of the implementation intervention and to highlight the distinctive ways that the intervention will address key context-specific behavioural issues.^22^

**Table 2:** Guiding principles for the development of the PADL implementation intervention

Design objectives	Key features of the implementation intervention
To motivate healthcare workers to take a penA focused history, risk assess penA histories and de-label those with a low-risk penA history.	Provide evidence on the positive impact of PADL on patient care. Provide evidence on the safety of PADL for patients. Promote PADL as a hospital-endorsed and senior clinician-endorsed endeavour through publication of hospital-approved guideline championed by senior clinicians. Provide opportunity to rehearse PADL through role play.
To provide easily accessible tools that facilitate PADL.	Provide guideline with the tools required to PADL.
To ensure the PADL process is delivered in a structured way to reduce relabelling.	Provide education on how to deliver PADL and provide the necessary tools.
To support patients with a low risk penA to agree to de-labelling and to take penicillin antibiotics as prescribed.	Make available patient information leaflets that HCWs can share on the risks and benefits of PADL.

PADL, penicillin allergy de-labelling; HCWs, healthcare workers; .

The guiding principles focus on motivating HCWs to deliver PADL through increasing confidence through training, provision of an evidence-based patient pathway and providing senior hospital clinician and hospital management support for PADL. Patient education was also a key intervention objective and that was met through provision of patient information leaflets (PILs) and HCW education on delivery of this important component of the intervention.

## The implementation plan

### Stage 3: optimising the implementation intervention

#### Stakeholder and expert workshops

##### Methods

Five workshops were convened via Microsoft Teams to discuss the phases of implementation plan (figure 2) and the intervention materials (table 3).

**Table 3:** Intervention materials for penicillin allergy delabelling implementation intervention

Intervention materials	Description	Purported mediators
For Pharmacists, doctors and MOPTs	The competencies covered in the slide set are as follows: Understand the consequences of an incorrect penA record. Understand drug allergy phenotypes and to differentiate these from intolerances (side effects that result in avoidance). Know which allergy phenotypes are eligible for de-labelling. Know which allergy phenotypes are not eligible for de-labelling. Understand the requirements of a penA focused history. Know how to risk assess penA histories using a decision support tool. Know the de-label methods and processes. Understand the risk of future reactions in low-risk patients. Information to patients Understand that PADL is done within a governance framework.	Increase knowledge, skills, confidence to take a penA focused history, risk assess, de-label and prescribe penicillin in a well-supported environment.
Education and training Part 1–20 min education and training slide set for doctors and pharmacists, delivered either as a recorded online slide set accessible via the hospital intranet, or face-to-face at education meetings. A posteducation test using Google Forms survey to ensure knowledge acquisition prior to moving to part 2.		
Part 2–Ward-based face-to-face competency-based training exercise, assessed by a penA champion.	Three role-played case studies. The learner taking the HCW role and the assessor the patient role. The assessor is to use a competency framework to score the learner.	
Part 3–First three DOC decisions to be peer reviewed by PADL champion.	Learner contacts a named PADL champion to discuss cases they believe are eligible for DOC prior to prescribing DOC test dose.	
Part 4–PADL champion assessor signs off learner if deemed safe to do so.	PADL assessor sign-off when competencies are demonstrated, and the learner is deemed safe to deliver PADL.	
Hospital PADL guidelines are published on the hospital’s intranet with a link in Microguide.	Contains: penA focused questions to ask patients How to document the answers to the questions in the EPMA system The steps in the DDL and DOC process DOC testing protocol GP letters to communicate new de-labelled status or to confirm drug allergy status.	Increase knowledge, skills and confidence to take a penA focused history, risk assess, de-label and prescribe penicillin in a well-supported environment.
A decision tool to aid risk assessment and de-labelling decision making	A validated decision support tool within the hospital guidelines used to risk stratify penA history obtained from the patient	Increase confidence to risk assess penA records.
Patient information leaflet-1 (pre-de-label counselling points) and patient leaflet-2 (what a negative DOC means)	Both leaflets, included in the hospital guidelines, contain the key counselling points for patients; acting as an aid memoir for healthcare workers and written information for patients to take away for future reference.	Patients motivated to undergo testing and reassured by the safety of PADL.
A PADL champion to attend existing specialty medical, surgical and senior nurse meetings to raise awareness about PADL and get support for PADL from senior specialty doctors and nurse matrons and ward sisters.	The key benefits of PADL and risks of PADL will be communicated at these sessions in a 10 min verbal presentation.	To increase confidence in the safety of PADL and to raise awareness of the importance of PADL. To ensure that senior clinicians are aware that PADL is supported by the organisation.
Produce an A4 information leaflet for ward sisters to communicate key messages about PADL and challenge testing observations at nurse meetings, nurse handovers and displayed in nurse communal areas.	Ward sisters are to communicate key messages about PADL and challenge testing observations at nurse meetings, nurse handovers and displayed in nurse communal areas.	To increase confidence in the safety of PADL and to raise awareness of the importance of PADL. To ensure that nurses are aware that PADL is supported by the organisation.
For patients	Description	
Patient leaflet-1	Patient information leaflet, given to patients by healthcare workers delivering PADL, that communicates the key risks and benefits of PADL to patients. Complements the verbal counselling provided by HCWs on risks and benefits of PADL.	Patients motivated to undergo testing and reassured by the safety of PADL.
Patient leaflet-2	Patient information leaflet, given to patients by healthcare workers delivering PADL, providing written information on what a negative test means for patients.	Patient reassured by the negative test result and confident in their new de-labelled status so that they no longer continued to avoid penicillin.
Expert leadership	Description	
Consultant antimicrobial pharmacist/lead AMS pharmacist and infectious diseases consultant available for penA advice.	Local experts in PADL will be available to offer advice to healthcare workers on any component of PADL, should the need arise.	Increase knowledge, skills and confidence to take a penA focused history, risk assess, de-label and prescribe penicillin in a well-supported environment.
Consultant antimicrobial pharmacist/lead AMS pharmacist and infectious diseases consultant to visibly champion PADL.	PADL will be promoted at ward level by the PADL champions while delivering infectious diseases, antimicrobial stewardship, or general medical ward rounds. This will include promoting and signposting ward healthcare workers to the PADL training, prompting PADL for patients who would benefit from PADL and supporting ward HCWs with delivering PADL.	Increase knowledge, skills and confidence to take a penA focused history, risk assess, de-label and prescribe penicillin in a well-supported environment.

AMS, antimicrobial stewardship; DDL, direct de-label; DOC, direct oral challenge; EPMA, electronic prescribing and medicines administration; GP, general practitioner; HCWs, healthcare workers; MOPT, Medicines Optimisation Pharmacy Technicians; PADL, penicillin allergy de-labelling.

Two 60 min workshops were convened with two implementation science experts from NIHR Peninsula Applied Research Collaborative (penARC), neither working at the study hospital, to discuss the intervention implementation plan.

Two 90 min workshops were convened with representation from innovation (PADL) experts, with either experience of delivering PADL in UK hospitals or experience delivering PADL research and local HCWs. Topic experts were from outside the study hospital, all but one were from the UK, and included five doctors or pharmacists with both research experience and experience delivering PADL, four doctors or pharmacists with research experience only and a nurse and pharmacist with experience delivering PADL only. Stakeholders were all from the study hospital and included two junior doctors, six medical consultants of which one was a medical microbiologist and two pharmacists. Feedback was sought on the implementation innovation plan and supporting materials.

The education and training materials and the training dissemination plan were reviewed by eight members of the University of Exeter’s Health Professions Education and Wellbeing research group (one 90 min workshop).^33^ Two junior doctors, two non-medical prescribers (one colposcopist and one paramedic) and three pharmacists were invited to complete the PADL learning material and provide feedback on each section via a proforma in Microsoft Word and verbally.

Verbal consent was obtained from all workshop participants and only written notes were taken. Participants were not reimbursed for participation.

The three PILs: (1) the risks of incorrect penA records and the risks and benefits of testing, (2) the information after a negative test and (3) information after a positive test) were emailed to four invited patients who had identified themselves as having a penA record. Feedback was sought on how easy the leaflets were to read and to understand, any words or sentences that were unclear, how reassuring the leaflet was for patients that PADL is safe, how persuasive the leaflet was and whether it would encourage them to get tested and invited any further comments.

##### Results

###### The NIHR Peninsula Applied Research Collaborative (penARC)

The group felt the intervention was comprehensive and rooted in the evidence from the qualitative studies. The group suggested consideration be given to the Consolidated Framework for Implementation Research (CFIR) outcomes addendum and Reach Adoption Implementation Maintenance Qualitative Evaluation for Systematic Translation (Re-AIM QuEST) when evaluating the implementation outcomes, which have been adopted as suggested.^34 35^

###### Innovation (PADL) experts

For a full list of discussion points, suggestions and actions, see online supplemental appendix B. In brief, key discussion points included suggestions from several participants that delivering the whole PADL pathway will be challenging for one HCW to deliver due to time constraints, but decoupling the PADL pathway into two deliverable sections would make it more accessible for ward staff. Part 1 might include documentation of a structured penA focused history that is easily accessible and part 2 includes the risk assessment and de-label. Part 1 would be accessible to all HCWs working on the ward while part 2 would be accessible to selected staff and delivered by those trained in PADL. Medication reconciliation is undertaken by pharmacy ward staff during which it was proposed that an allergy history could be taken; PenA history taking embedded in that process, and ensuring senior pharmacist buy-in to PADL, should facilitate MOPT and ward pharmacist uptake of penA history taking.

Education was suggested to be key to the intervention implementation, but time constraints would hinder adequate education dissemination. Separating the education into two parts; part 1, awareness raising which includes how to take a penA history and risk assess patients, and then part 2, further cases-based training on risk assessment and the de-label process, would facilitate delivery. Part 2 could be offered to ward pharmacists and junior doctors as part of their development and would enable them to champion PADL in their clinical areas.

Communicating to HCWs that penA records create health inequality and that not delivering PADL means patients receive second line care and taking key messages about the benefits of PADL to senior medical, nursing and pharmacy meetings for discussion and endorsement would ensure senior support and facilitate PADL in clinical areas.

Modifications to the exclusion criteria for DOC were suggested which included removing haemodynamic parameters outside normal range as an exclusion criterion but clarifying that haemodynamic instability and clinically deteriorating patients were to be excluded. Low, but stable, oxygen requirement was changed to ‘caution’ instead of a contraindication for DOC, and a suggestion to use clinical discretion for these exclusion criteria.

###### Education and training material

Overall, the group felt that the education material was a great example of an educational intervention with clear clinical relevance. Some of the suggested modifications were actioned: MCQs were edited to reduce the cognitive load and increase ease of reading for the learner and an extra question was added to explore learning knowledge about the range of allergy phenotypes; acronyms in the recorded slide set were written in full; competency-based assessment marking criteria were modified to include the GP communication domain; and, the clinical information in the case studies was edited to make them succinct. It was noted that the purpose of the clinical case studies was not clear to the learner and so an instruction for learners’ paragraph was added that preceded the case study (see online supplemental appendix C for full feedback and rebuttal).

The learning package was well received by the reviewing junior doctors, non-medical prescribers and pharmacists without suggestions for improvement except for minor edits to the text.

###### Patient and public involvement and engagement review of participant information leaflets

The leaflets were broadly well received and described as interesting, informative and very easy to understand. There were several suggestions made by the four PPIE members that were acted on. These included making some edits to the leaflets to ensure all the language was patient friendly; to address the fact that this is going against previous medical advice where patients with penA have always been told to avoid penicillin; to explain in the leaflets what has now changed and also make it clear in the leaflets that PADL is a choice for patients; and, some reassurance about what would happen should they have a reaction to penicillin (see online supplemental appendix D for full list).

### Stage 4: outcome measures

#### Methods

By discussion within the core team, we used the CFIR outcome addendum to identify implementation intervention (termed implementation in the CFIR) and PADL intervention outcomes (termed innovation in the CFIR).^34^ The (CFIR) Outcomes Addendum groups implementation outcome measures into three groups: adoption, implementation and sustainment and also has one innovation outcome (PADL) group. These are mapped to both the Reach, Effectiveness, Adoption, Implementation and sustainment (RE-AIM) and the Implementation Outcomes Framework.^36 37^ Measurement of both the intervention outcomes (see figure 1 (logic model) PADL and penicillin prescribing) and the implementation intervention outcomes (see online supplemental appendix E) are required to evaluate the success of this implementation intervention.^34^

The implementation intervention outcomes (online supplemental appendix E) will provide information about whether the intervention changes HCW and patient behaviours and which determinants within the implementation intervention are successfully delivered and which were not and why they were a success or not. Collecting these data will increase knowledge about what works in each setting and can be used to modify the intervention to further increase adoption so that it becomes integrated into routine clinical practice locally and used to facilitate implementation in other UK National Health Service settings.

The intervention outcomes will provide information about the patient level impact of PADL to determine whether PADL (delivered by a variety of HCWs: ward clinical teams; doctors, pharmacists and nurses and antimicrobial pharmacists) is safe and whether PADL influences the types of antibiotic prescribed. The number of patients de-labelled over time, if an increasing trend, will provide an indication as to whether the intervention is changing HCW and patient behaviours around PADL.

#### Results

The findings of this process are described in online supplemental appendix E.

## Discussion

In this paper, we describe the development of an implementation intervention that aims to change the behaviours of patients and non-specialist HCWs to widen delivery and acceptance of PADL services in a district general hospital. We combined evidence from qualitative research and behaviour theory to design the implementation intervention and refined it iteratively through engagement with stakeholders and experts in the field. PADL is expected to have a positive impact on AMS through increased use of narrower spectrum penicillin antibiotics and concomitant reductions in broader spectrum agents. We identified outcome measures that would both capture the effectiveness and safety of the intervention and measure the fidelity of the planned implementation strategy. This approach to intervention design has been used by us and others to develop AMS interventions, including a PADL intervention in a different setting.^23^,^25^ Our PADL implementation intervention has similarities to the antimicrobial review kit study, an intervention that safely reduced antibiotic use in hospitals, which also identified the need for champions to lead the implementation, the need for wide clinical stakeholder engagement, education for HCWs, information for patients and a decision support tool.^23^ There were also similarities with ALABAMA, a PADL study initiated in primary care, which similarly identified the importance of robust communication with patients and between secondary and primary healthcare settings.^24^

We drew on the PBA because of its focus on stakeholder engagement and codesign with target users, which is expected to enhance local uptake of PADL by making the implementation intervention more locally relevant, acceptable and feasible. This approach also aligns with the MRC’s framework for developing and evaluating complex interventions.^19^

We, and others, have identified significant barriers to delivering PADL, including the time required to deliver PADL and a lack of confidence in being able to deliver PADL by some junior doctors and pharmacists.^31 38 39^ Addressing these barriers is important if this intervention is to be successfully implemented.^40^ Confidence will be addressed through completion of the training module; however, the issue of time may be a barrier to undertaking training. The PADL champions will be supporting the delivery of PADL in clinical areas which is expected to increase HCW motivation and confidence to deliver PADL. To address the issue of time, we will present PADL to HCWs as a two-stage process; first, penA history taking and documentation, second penA risk categorisation and de-label. In this way, part 1 could flag patients for somebody else with PADL expertise to de-label the patient later, as capacity allows. PenA history taking will be incorporated into the medication reconciliation process.

Likewise, time to undertake the requisite training for PADL is likely to be a significant barrier to implementation of the intervention. Consequently, we split the training into a two-part process to make it more accessible and deliverable; part 1 a recorded or face-to-face slide set covering the background to PADL and the PADL processes, and stage 2a case-based ‘rehearsal’ of PADL delivered face to face to learners by PADL champions.

Blumenthal *et al* described their experiences of designing and delivering a ward-based clinical team-delivered PADL intervention across five hospitals in the Boston area in the USA, which included the formation of an implementation team, their selection of a PADL approach (guidelines and decision support tool), wide stakeholder engagement (including hospital leadership, infection specialists, pharmacy and nursing) and spreading the change. through education and establishing measures and evaluating impact; taking a similar approach to the implementation intervention described here.^41^ There are some notable differences. Although our PADL guideline has been approved by an executive (medical director) and supported by senior medical, surgical and pharmacy staff, we do not have an executive sponsor for our PADL programme of work, but part of the guideline approval process we agreed to provide feedback on the intervention to the hospital’s Medicines Practice Committee and as such an executive does have oversight of the PADL programme. Blumenthal *et al* were able to add a best practice advisory (BPA) to their electronic prescribing system that alerts when prescribing non-beta-lactams for patients with a penA and prompts HCWs to review the penA. Our EPMA system does not have BPA but we do have a surveillance system that enables the AMS team to identify such patients. Blumenthal *et al* describe an education programme that is both succinct and flexible, with multimodes of HCW education available through their intranet and face-to-face and have included PADL as part of mandatory training. Our education strategy consists of both face-to-face and e-learning and is targeted to different audiences, but it is not available via the intranet. Plan-Do-Study-Act cycles were set up to review frequency of test doses and patient safety, and these data were interrogated so that they could further improve the intervention, a process similar to our planned evaluation. We have taken this further by adopting the RE-AIM QuEST framework, a co-ordinated use of quantitative and qualitative measures to further explore barriers and enablers to adoption of the intervention with end users, the findings of which will inform iterative improvements to further optimise the implementation intervention so as to enhance sustainability within the study hospital and to understand potential barriers and enablers to translation to other settings.

### Strengths and limitations of this study

We followed a systematic process to development of the intervention using the views of local patients and local HCWs and behavioural theory. We engaged large and diverse stakeholder groups to refine the intervention which included educationalists, topic experts with practical and research experience with delivering PADL and predominantly senior clinicians.

There were few pharmacists, MOPTs and nurse stakeholders in the refinement stages, but we feel we had reasonable representation from these groups in the predevelopment stages in the qualitative studies. We had patient involvement in refining the PILs only and not the implementation intervention. But again, we had good patient representation in the predevelopment qualitative study.

## Conclusions

We have described the development of theory-based and stakeholder-developed implementation intervention designed to support inpatient PADL delivered by a multiprofession workforce. The intervention will be implemented in a single hospital and evaluated in an implementation study with the outcomes used to further optimise the intervention and explore scalability.

## Supplementary material

10.1136/bmjopen-2024-096452 online supplemental file 1

## Data Availability

Data sharing not applicable as no datasets generated and/or analysed for this study.
