# Supplementary material for: Description of the combined evidence-based, theory-based and person-based approaches used to develop a behavioural intervention package to support non-allergist healthcare workers to remove incorrect penicillin allergy records from medical and surgical adult inpatients in a UK hospital
Source: BMJ Open. 2025 Jul 30;15(7):e096452. doi: 10.1136/bmjopen-2024-096452 (PMC12315033; doi:10.1136/bmjopen-2024-096452)
Supplement: online supplemental file 1 [file bmjopen-15-7-s001.docx]

**Appendix table A1 Behaviour analysis table for REPeAL IS intervention, healthcare worker behaviours. HCW, healthcare worker; PenA, penicillin allergy, MOPT, medicines optimisation pharmacy technician; PADL, penicillin allergy de-labelling; DOC, direct oral challenge;**

| **Barriers / facilitators to**  **target behaviours** | **Intervention**  **Component/s** | **Intervention ingredient** | **Theoretical Domains**  **Framework (TDF) V2** | **Target**  **construct**  **(BCW)** | **Intervention**  **function (BCW)** | **Behaviour Change Technique (using 93 BCT taxonomy v1) (BCW)** |
| --- | --- | --- | --- | --- | --- | --- |
| **Target behaviour: Pharmacists, doctors and MOPTs to take a penA allergy focused history on admission / during inpatient stay to hospital** | | | | | | |
| HCWs are not confident taking a penA focused history. | 20-minute education & training module to include how to take a penA focused history. (Delivered either as a recorded online slide set, accessible via the hospital intranet, or face-to-face).  Ward-based competency-based training module, delivered by a penA champion, to include taking a penA focused history.  Hospital guidelines and Microguide to list the penA focused questions that need to be asked during PenA history. | Provide information on the questions to ask, the rationale for questions and where to locate the questions for future reference (in the hospital PADL guidelines/ Microguide).  Provide opportunity to rehearse penA history taking with feedback from trainer.  Make the penA questions accessible to HCWs. | ↑ knowledge  ↑ skills  ↑ beliefs about capabilities  ↑behavioural regulation  ↑environmental context & resources  ↑Memory, attention and decision process | -Psychological capability.  -Reflective motivation  -Psychological capability.  -Physical opportunity | Education, enablement,  Training, enablement  Environmental restructuring | 4.1. Instruction on how to  perform the behaviour.  5.1 information about health consequences  8.1 Behavioural practice/ rehearsal  12.5. Adding objects to the environment |
| PADL is not viewed as a priority. | 20-minute education & training module to include information on the risks of penA labels.  Consultant Antimicrobial Pharmacist / Lead AMS pharmacist and Infectious Diseases consultant to visibly champion PADL.  Senior support is required for more junior staff to de-label (DOC).  Produce, publish, and publicise a hospital endorsed PADL guideline. | Provide information on the negative consequences of penA records.  To promote PADL and actively provide peer support and advice to ward based HCWs.  To provide evidence of hospital leadership support for PADL.  To provide demonstrable organisational support for PADL | ↑Intentions  ↑Optimism  ↑Knowledge  ↑behavioural regulation  Knowledge; beliefs about capabilities, reinforcement, intentions, memory, attention & decision-making process, environmental context & resources, social influences, behavioural regulation, professional role & identity.  Social/professional role & identity, intensions, environmental context & resources, social influences; behavioural regulation  Environmental context & resources, social influences. | -Reflective motivation  -Automatic motivation  Social opportunity; Reflective motivation  Social opportunity  Physical opportunity, social opportunity, | Incentivisation, persuasion, education,  Modelling, enablement, persuasion,  Persuasion, enablement, modelling  enablement | 5.1. Information about health consequences  6.1 Demonstration of the behaviour  3.1. Social support (unspecified)  3.2. Social support (practical)  9.1 credible source  7.1. Prompts/cues  9.1. Credible source  3.3. social support (emotional/ unspecified)  6.3 information about others’ approval  3.2. Social support (practical) |
| **Barriers / facilitators to**  **target behaviours** | **Intervention**  **Component/s** | **Intervention ingredient** | **Theoretical Domains**  **Framework (TDF) V2** | **Target**  **construct**  **(BCW)** | **Intervention**  **function (BCW)** | **Behaviour Change Technique (using 93 BCT taxonomy v1)** |
| **Target behaviour: Pharmacists, doctors and MOPTs to risk assess the penA history.** | | | | | | |
| Not able to confidently differentiate low risk and high-risk penA histories. | 20-minute education & training module to include risk assessing penA histories with a decision support tool and explanation on which patients can be safely de-labelled by DDL/DOC.  Ward-based competency-based training module, delivered by a penA champion, to include how to risk assess penA histories, and to decide de-label method, using a decision support tool.  Hospital PADL guidelines to include a decision support tool. | Provide the knowledge on how to use the decision support tool to risk stratify patients and to decide de-label method.  Provide opportunity to rehearse risk assessing penA histories and using a decision support tool to decide de-label method.  Provide a validated decision support tool to risk assess penA histories and decide de-label method. | ↑Knowledge  ↑social/professional role & identity  ↑Intentions  ↑behavioural regulation  ↑Skills  ↑beliefs about capabilities  ↑memory, attention & decision processes.  ↑environmental context & resources | -Psychological capability.  -Reflective motivation  -Psychological capability.  -Physical opportunity | Education, enablement  Training, enablement  Enablement, environmental restructuring | 2.7. Feedback on outcome(s)  of behaviour  2.3. Self-monitoring of  Behaviour  1.2. Problem solving  8.1 Behavioural practice/ rehearsal  1.2. Problem solving  12.5. Adding objects to the environment |
| **Target behaviour: Pharmacists and doctors to either de-label on history alone or prescribe direct oral challenge dose** | | | | | | |
| Pharmacist & doctor concerns about the safety of DOC. | 20-minute education & training module to include information on the safety of DDL/DOC in low-risk patients.  Ward-based competency-based training module, delivered by a penA champion, to include peer supported decision making with the first few decisions to DOC discussed with PADL champions prior to competency sign-off | Provide evidence for the safety of DOC/DDL in low-risk patients.  To increase HCW confidence with safely de-labelling via DOC. | Knowledge; optimism, beliefs about consequences, emotion.  Skills, optimism, beliefs about capabilities, environmental context & resources, social influences. | Psychological capability, reflective motivation.  Physical capability,  automatic motivation. | Education  Training, enablement | 5.1. Information about health  Consequences  8.1. Behavioural rehearsal / practice 11.2 reduce negative emotions |
| Senior support is required for more junior staff to de-label (DOC). | A PADL champion to deliver the 20-minute education & training module face-to-face at all specialty governance or education meetings.  20-minute education & training module to provide evidence of senior pharmacy, medical & nursing leadership endorsement of PADL. | To provide information on the rationale for PADL to ensure specialty support for PADL in their specialties.  To provide evidence of hospital leadership support for PADL. | Knowledge; optimism, beliefs about consequences.  Social/professional role & identity, intensions, environmental context & resources, social influences; behavioural regulation. | Psychological capability,  Social opportunity reflective motivation.  Social opportunity | Education  Persuasion, enablement, modelling | 9.1. Credible source  5.1 information about health consequences  9.1. Credible source  3.3. social support (emotional/ unspecified)  6.3 information about others’ approval |
| Pharmacists and doctors would like access to an expert for advice when required. | Consultant Antimicrobial Pharmacist / Lead AMS pharmacist and Infectious Diseases consultant available for penA advice. | To provide expert advice when required with decision making around DOC/DDL. | Skills, social/professional role & identity, beliefs about capabilities, environmental context & resources. | Social opportunity | enablement | 3.3. social support (practical)  1.2. Problem solving |
| *A PADL champions would optimise PADL.* | Consultant Antimicrobial Pharmacist / Lead AMS pharmacist and Infectious Diseases consultant to visibly champion PADL.  Infection specialists to champion PADL while giving infection advice and reviewing patients with infectious diseases. | To promote PADL and actively provide peer support and advice to ward based HCWs.  To prompt PADL when giving advice if clinically indicated. | Knowledge; beliefs about capabilities, reinforcement, intentions, memory, attention & decision-making process, environmental context & resources, social influences, behavioural regulation, professional role & identity.  ↑Intentions, ↑environmental context & resources, ↑ social influences | Social opportunity; Reflective motivation  -Reflective motivation  -social opportunity | Modelling, enablement, persuasion,  Environmental restructuring, enablement | 6.1 Demonstration of the behaviour  3.1. Social support (unspecified)  3.2. Social support (practical)  9.1 credible source  7.1. Prompts/cues  3.1. Social support (unspecified) |
| PADL is not currently supported by the organisation. | Produce, publish, and publicise a hospital endorsed PADL guideline. | To provide demonstrable organisational support for PADL. | Environmental context & resources, social influences. | Physical opportunity, social opportunity, | enablement | 3.2. Social support (practical) |
| **Barriers / facilitators to**  **target behaviours** | **Intervention**  **Component/s** | **Intervention ingredient** | **Theoretical Domains**  **Framework (TDF) V2** | **Target**  **construct**  **(BCW)** | **Intervention**  **function (BCW)** | **Behaviour Change Technique (using 93 BCT taxonomy v1)** |
| **Target behaviour: Nurse to perform baseline observations and post oral challenge test observations.** | | | | | | |
| There needs to be senior nurse support to deliver target behaviour. | Awareness raising about the benefits and the safety of PADL delivered face to face by antimicrobial stewardship pharmacists to all senior nurses delivered at matron and ward manager meetings. | To provide information to senior nurses on the risks of penA records.  To provide evidence on the safety of DDL/DOC in low-risk patients | Knowledge, social influences | Social opportunity | Enablement, | 3.1. Social support (unspecified)  6.3 information about others’ approval |
| There are competing demands on time. | Produce an A4 information leaflet to be used by for ward sisters to communicate key messages about PADL and challenge testing observations at nurse meetings, nurse handovers and displayed in nurse communal areas. | To provide information to ward nurses on the risks of penA records and on the safety of DDL/DOC in low-risk patients.  To give permission to ward nurses to delegate, or to share the responsibility to deliver the observation with others (HCA, med/nurse students, others). | Knowledge, professional role & identity, beliefs about capabilities, | Physical opportunity, Reflective motivation | Education, enablement | 1.2. Problem solving  5.1. Information about health consequences |
| **Target behaviour: Pharmacists and doctors to counsel the patient on the risks of penA records and on the risks and the benefits of PADL.** | | | | | | |
| Poor patient counselling by doctors and pharmacists on the risks and benefits of PADL will reduce patient uptake of PADL. | 20-minute education & training module to include key counselling points and the importance of discussing these key points with patients.  Ward-based competency-based training module, delivered by a penA champion, to include key counselling points. | To provide information on the key patient counselling points.  To understand the importance of patient counselling on optimising patient uptake of PADL.  To rehearse providing pertinent counselling information to patients. | Knowledge, beliefs about capabilities,  Knowledge, intentions, optimism  Skills, beliefs about capabilities, | Psychological capability,  Psychological capability, reflective motivation,  Psychological capability, | Education, enablement  Education, persuasion,  Education, enablement, training, | 5.1. Information about health Consequences  5.2. Salience of consequences  4.1. Instruction on how to  perform the behaviour |
| Counselling information needs to be accessible at the point of need for pharmacists and doctors. | PIL within the hospital PADL guidelines. | To make PILs accessible to HCWs via the intranet at the point of need to facilitate delivery of key counselling points. | Environmental context & resources. | Physical opportunity, | Environmental restructuring, enablement | 12.5 Adding objects to the environment |

**Appendix A Table A2. Behaviour analysis table for REPeAL IS intervention, patient behaviours.**

| Barriers / facilitators to  target behaviours | Intervention  Component/s | Intervention ingredient | Theoretical Domains  Framework (TDF) V2 | Target  construct  (BCW) | Intervention  function (BCW) | Behaviour Change Technique (using 93 BCT taxonomy v1) |
| --- | --- | --- | --- | --- | --- | --- |
| **Target behaviour: Patient to accept offer of de-label via either DDL or DOC** | | | | | | |
| Concerns about safety of testing, particularly amongst patients with advanced age, comorbidities, or high acuity illness. | Patient leaflet.  HCW training. | Provide information about safety of test.  Provide reassurance about the safety of PADL in advanced age, higher acuity illness and comorbid patients. | Knowledge, emotion, beliefs about consequences. | Psychological capability, reflective motivation, | Education | 5.1. Information about health  Consequences.  6.2 Social comparison  9.1. Credible source |
| Unaware of the negatives of taking alternatives to penicillin. | Patient leaflet  HCW training | Provide information on the negative consequences of avoiding penicillin. | Knowledge, beliefs about consequences. | Psychological capability, reflective motivation, | Education | 5.1. Information about health  Consequences.  6.2 Social comparison  9.1. Credible source |
| **Target behaviour: (be willing) to take penicillin when prescribed** | | | | | | |
| *De-labelled patients had confidence in the PADL pathway and the negative test result.* | Patient leaflet 2  HCW training | Provide confirmation to patient that they can take penicillin safely | Beliefs about consequences | Reflective motivation. | Education | 5.1. Information about health  consequences |

**Appendix B summary of Person Based Approach expert stakeholder panel member discussions, suggestions and actions.**

| **Summary of main points** | **To do to intervention (if anything)** | **Actioned?** |
| --- | --- | --- |
| Add PADL guidelines to the ‘Health Toolbox’. | - Find out what the Health Toolkit is. - Find out who owns it so that I can add PADL guidelines to it. | No, kept the guidelines in the ‘documents library’ in intranet so that they are not in multiple places (issues with version control) |
| Asking consultants to PADL is not going to be deliverable and asking clinical teams to do it in ED/AMU is a challenge due to competing priorities. But, penA hx taking and identifying potential patients is possible in ED/AMU and by consultants. | Make it clear that PADL is a two-step process, and that step 1 is quick and easy. | Yes |
| Opportunity and motivation are both challenges, capability less challenging | To explore how to make PADL more accessible to HCWs and to motivate them to be engaged with PADL through face-to-face education sessions and the planned semi-structured interviews at weeks 12-24. | Yes |
| PenA is a health inequality. This should be a hospital focus. Sell the benefits (pt, LOS, ££, AMR, mortality, morbidity) vs risk based on assessment. | - Seek support/endorsement/recognition from pharmacy leaders. - Seek support/endorsement/recognition from medical leaders. | Yes  Yes |
| Consider PADL as a KPI for specialties | No, feedback from qualitative studies was to not do this | No |
| Provide written information to patients to evidence de-label that can be used to communicate de-label status to other healthcare providers. | Already part of PADL implementation intervention | Yes |
| Provide face to face PADL education as well as recorded option. | Both modalities will be offered to learners | Yes |
| Training needs to be in chunks because getting an hour of staffs’ time will be a challenge. Consider increasing the length of training. | Training chunked in to two discrete modules | Yes |
| We are embedding best practice and should be doing this anyway. PADL needs to be embedded as a standard of care and ongoing AMS intervention | Make it clear in the education module that PADL is best practise. | Yes, part 1 training and promoted when engaging with specialties at governance meetings. |
| Not doing PADL means we are providing second line care with drugs that are very broad spectrum, more toxic and negative impact on society | Make it clear in the education module that not delivering PADL means you are providing second line care for your patient. - creates inequality of care | Yes, part 1 training and promoted when engaging with specialties at governance meetings. |
| Consider tailored training to different staff groups, e.g. pharmacists and ED consultants have differing learning needs. The emphasis on elements of the training will likely need to differ between these groups of staff. | Consideration given to the content in part 1 and part 2 and who the intended audiences are for each/both | Yes |
| Sign post learners to further reading. | Add further reading to the slide set.   - BSAC MOOC - Lancet paper | Yes (part 1 education) |
| Consider the training in podcase format as well as a slide set. | Potential to miss learning with absence of visuals too? | No |
| Split PADL in to two processes; (1) history taking and then (2) de-label. All HCWs can for 1 and train people to safely do 2. The easy patients can be de-labelled in part 1 (the intolerances and those who have tolerated subsequent exposure can be dealt with) and the DOCs will be dealt with by somebody else; some AMS team and some ward docs, if trained. | Adopt this mechanism and promote this mechanism in all communications and training about PAD inpatient pathway. | Yes |
| Train those that will de-label and educate/awareness raise to those that might ask the ques and determine risk. No point training everyone to PADL. | Two sets of education – (1) awareness raising that includes how to take a penA hx, risk assess and flag and (2) the full training with PADL | Training in two parts |
| Ensure MOPTs are trained to take a penA hx because they are very involved in the medication reconciliation process | Get the ESR module as part of MOPTs training. | Yes, all MOPTs to get part 1 PADL training which includes how to take and document a penA history |
| Really keen juniors might volunteer for training and being PADL champions. Train as many junior doctors as you can. Consider making it a half day training with study leave. That would be good CV fodder for them, and they will be more motivated to squeeze a PADL in between the other work. Also, ward pharms, similar, find some champions/enthusiasts to train. | Put all junior doctors and ward pharmacists through part 1 training and offer part 2 at those sessions. | Yes |
| Pharmacists are the obvious group of HCWs to train up to PADL. Asking all clinicians to do the training is going to be challenging to implement. | Get PADL as part of all pharmacists and MOPTs training | To set up during implementation stage. |
| If there are staff able to de-label, then more senior docs will more likely take a penA focused history if they know it will be actioned. | Could ask the consultants to encourage their ward pharms and junior docs to be signed off and engaged in PADL. | To request that support from senor clinicians in the speciality engagement sessions. |
| Putting the 20-minute slides into mandatory training for consultants would be a good way to get that in front of consultants- a good way to embed the seed of the idea of PADL. In that, the pathway can be explained, i.e. that risk assessing and flagging it will be picked up by somebody (ward pharm, ward doc, AMS team). | Include some PADL awareness raising in consultant mandatory training.  Explain the PADL pathway in that training. | Added to consultant training slide set |
| Suggestion that maybe mandatory training isn’t the way to go as mandatory training isn’t popular and it may dissuade people. A Grand Round and then champions supporting/promoting it and seeding PADL in that way. The AMS team and champions doing it then that may be a good way to spread PADL. | Find champions to train up and to spread PADL education and awareness through other teaching opportunities. | Plan to recruit champions at specialty meetings, education meetings, and on PADL ward rounds |
| Report de-labelling metrics at governance meetings i.e. how many low risk were de-labelled to demonstrate something is being done about patients flagged by consultants and that will be motivating for consultants and garner buy in. | Consider reporting PADL metrics at governance meetings to motivate others to be involved. | To consider as part of implementation plan. |
| Presenting case studies where PADL made a positive difference to patient care would be motivating for doctors to get involved in PADL – find examples and present these at meetings. | Include case studies in any face-to-face training/ education where PADL had a positive impact on patients. | To collate case studies for inclusion in training and education sessions. |
| Messages that ought to be communicated to HCWs  -Increased mortality in those with a penA (pneumonia and endocarditis data)  - penicillin allergy label is a health inequality and it needs the translation of increased mortality, extra financial costs, AMR increase use and watch and reserve antibiotics. | Try and translate the published data to RCHT patients and potential impact for RCHT of PADL – e.g. endocarditis and CAP mortality data. | To include in specialty meeting awareness raising and education material. |
| Penicillin allergy history taking needs to be part of the pharmacy medicines reconciliation process as a standard of care. | Ask senior pharmacy colleagues to endorse penicillin allergy history taking as standard of care and part of the medicine’s reconciliation process. | Seek chief pharmacist and clinical pharmacy manager support. |
| PADL is going to take time to embed and will be multipronged – education, audit feedback, benchmarking, case studies. etc etc | Acknowledge that the PADL embedding process will take time | Nothing |
| Feedback specialty specific / relevant information tailor to oncology, to haem, to peri-op etc. the benefits. Needs to be ongoing and re-iterated at these specialty meetings. Can then find champions within those teams doing it that way. | Ensure there is a plan for regular feedback to specialties. | Attempt to make specialty engagement sessions specialty specific, where able. |
| Competition between wards or specialties re PADL rates | Consider a league table between specialties but I will need to be mindful that in the qualitative interviews HCWs had expressed a wish that targets/performance metrics were not brought in for PADL. | Consider how to do this. Specialty specific PADL figures may be more appropriate. |
| We don’t want the work delivering PADL to be lost through non-updating GP records. We need to explore how best to communicate the message to primary care and then make sure it is acted upon. | Whether there is communication between secondary care and primary care, and whether GP records are updated are both outcome measures. | If communication from secondary care is poor or GP record updates low, then an intervention to improve performance will be required. |
| As a follow up to PADL, monthly, we could tell GP surgeries all these patients listed with your surgery have had a penicillin allergy test so that it is all in one document for GP surgeries to check their records are updated. | I need to explore with GP staff how best to communicate PADL status with them. | Consider these once discussions have been had with GP surgery and we know how well penA records are being updated. |
| Put info about PADL in the discharge letter as an action for GP. A standalone clinical letter was developed in one hospital to try an attempt penA records to be updated. | - Made PADL an ‘action’ for GPs not just ‘info’ for GPs - Needs a standalone dedicated clinic letter. | A standalone letter and an action in the discharge letter are both options in the implementation intervention. Review most appropriate modality during implementation. |
| In another centre, many letters didn’t make it through the filtering process at GP surgeries. They gave the standalone letter to the patient and asked them to GP with the GP surgery that their record had been updated. | A patient information leaflet detailing the PADL process, and their new allergy status is part of the implementation intention package. | Nothing to do. |
| Suggestion that not just communication to GP surgeries about those patients successfully de-labelled, but also information should be passed to GPs about moderate and high-risk assessments too so that records can be updated. | This will increase the communication workload. To consider usefulness of this with GP surgery staff before bringing this into the implementation intervention. | Consider adding this in at a later stage. |
| **Second peer group** | **To do to intervention (if anything)** | **Actioned?** |
| Exclusion criteria – remove SCAR to non-penicillin. If somebody has SCAR to a drug that is drug specific. | Update the exclusion criteria for DOC to exclude history of SCAR to non-penicillin antibiotics. | Hospital guidelines reviewed and updated. |
| Start simple or start slow and then gradually as people gain confidence and were increasingly more aware of the protocol in the broader sense as well as their ability to manage that opening it up. | Ensure HCWs trained to deliver PADL do it at a small scale to start and are well supported. | Incorporated into part 2 of the training. |
| If haemodynamically stable but parameters outside the normal ranges, then ok to DOC. Not ok if truly has hemodynamic instability. | Update exclusion criteria so that HD instability is an exclusion (contra-indication) but parameters outside normal range is not (caution). | Hospital guidelines reviewed and updated. |
| Amend exclusion criteria to not exclude all patients on oxygen. Those on low oxygen, or weaning oxygen would be ok to test. Those with high oxygen requirements not. | Amend the oxygen exclusion criteria in the PADL guidelines. | Hospital guidelines reviewed and updated. |
| Need to think of DOC exclusion criteria as either absolute or relative contra-indications | Review exclusion criteria and move some to relative CIs. Or, add some nuance and clinician discretion. | Hospital guidelines reviewed and updated. |
| Caution advised with DOC in deteriorating patients in case the use of penicillin is attributed to the demise of the patient. | Add a line in the PADL guideline about not DOC patients who are clinically deteriorating and perhaps state why (in case in complicates things!). | Hospital guidelines reviewed and updated. |
| Review exclusion criteria at 6 months and remove some. | Consider removing some exclusion criteria after a 6-month review. | To do |
| To engage with specialties and to ensure their support for PADL it might be better to go to each individual speciality meeting rather than send an email. If an email were to be sent, then just go through clinical leads or clinical governance groups for discussion at governance meetings. | Email specialties to get their support for PADL. Surveys don’t get completed very well. | Evidence specialty support for PADL via email to specialty/ governance leads and consensus to support or not from each specialty. |
| Engage the heads of nurses and explore getting PADL on the clinical skills.net platform which the nurses use a lot of their Procedural stuff. For example, there is a sheet on cannulation, and other information for nurses. | Engage with heads of nursing about PADL plans and provide an information sheet on PADL for dissemination to ward nurses. | Engage HONs and ask about clinicalskills.net platform |
| Link adverse cases/events in with the patient safety Incident Response framework (PSIRF). If there is an adverse outcome, then link in with your quality and safety colleagues and clarifying their understanding of incident reports and management around this topic. | Talk to Medication Safety Officer and ensure their awareness of PADL. | Link in with MSO |
| Need to measure whether the PADL implementation intervention is normalising penicillin allergy assessment amongst clinicians and taking this approach more widely beyond inpatient areas of the hospital, i.e.  elective settings.  As an as a measure of sustainment, see how many people become more aware and more confident with the concept of de labelling | To explore this in the semi-structured interviews with patients and healthcare workers between weeks 12 and 24 post implementation. | Need to measure GP record update and whether they are still reflecting the allergy status at 12 months (perhaps). This is an outcome measure. |
| Re-labelling is a real problem. Look at the primary care records at six months or 12 months to see if the delaying status is persisting or not. | Looking at GP records to determine whether they have been updated is part of the planned outcome measures. | nil |

**Appendix C Education experts review of education package; their comments and study lead responses/actions**

| Feedback | Rebuttal / actions |
| --- | --- |
| MCQs:  There was a consensus that when constructing MCQ questions such as these, attention should be given to trying to reduce the cognitive load of the questions as much as possible. It was thought that this could be achieved in a number of different ways: |  |
| • Combining the bold heading with the question that followed (the bold heading appeared to signpost the type of knowledge being assessed – which felt unnecessary for the participants). | Thank you, now removed the learning objective. |
| • Considering stating how many correct answers there were for each question. | The questions become very easy if I do that, so not actioned. |
| • In question 8 the use of the word “key” before requirements felt unnecessary. Consider instead asking: “Which of the following are requirements for a safe de-labelling?” | Removed ‘key’ from the question |
| Questions 1 and 4 were slightly difficult to read. These could have been simplified by combining the bold statement and the question as suggested above. | Done. I have reworded que 1 |
| It was felt that the questions perhaps missed an opportunity to assess participant’s understanding of the range of allergic reactions beyond anaphylaxis. From our perspective this is something that is often poorly understood by junior (in particular foundation) doctors. | I have added an extra question (now que 3) which explores recognition of the range of genuine allergy phenotypes. |
| It was also suggested that it might be helpful to add an introductory sentence or two outlining the role of the MCQs (knowledge testing) as part of the wider educational package. | Now added. |
| We also wondered if these MCQs have been piloted on the target audience? This would help to determine how easy they were to understand, how long they took to complete etc. | Plan to pilot in the first cohort of learners. |
| Competency-based assessment template:  Overall it was acknowledged that the format and process of supporting HCWs to develop and demonstrate competence in this field was complicated and involved multiple interacting components (hence it being a complex intervention). It would have help us, and perhaps those being asked to complete this, if the process could have been made clearer. Perhaps through developing a flow chart to outline the components of the educational intervention and assessment.  It was noted that acronyms were included which were not immediately clear (DOC, DDL).  The framework did not include the need to demonstrate the importance of feeding back this information to the patient’s primary care provider, or the ability to distinguish between allergy and intolerance (e.g. diarrhoea). | To do a flow chart of the learner’s progress through the learning module |
|  | Acronyms now in full  A competency about GP communication now added to the competency form.  The distinguishing between allergy and intolerance (e.g. diarrhoea) in already there “To be able to differentiate low risk, high risk and intolerances from the allergy history using a decision support tool.” |
| Clinical cases (we focussed on case 1 in detail):  A lot of clinical information was provided, particularly in the information for the HCW. It was felt that perhaps the quantity of this information was excessive and possibly unnecessary if the purpose of the station was to demonstrate an ability to take a focussed history regarding penicillin allergy. It was also felt that removing some of this detail might make the station feel more relevant to more of the target audience who are likely to be working in different settings.  We noted that after the initial narrative information a list of questions was included, presumably that the HCW was expected to ask? It was felt that perhaps these should not be given to the professional, or at least not given in this format. Would you expect a professional to know these questions without being told them?  In the instructions the specific purpose of the clinical simulated scenario was not clear. Would you consider providing more specific details of the task at the top of the scenario. For example: “Please read the following information and then take a detailed history to determine if…. Please explain the next steps to the patient. You have x minutes to do this”.  It was noted that in the patient instructions for case study 1 there were limited instructions for the actor regarding the patient view. From our experience patients often have a clear opinion regarding whether or not they have a penicillin allergy, even if there is no biological evidence to support their view. There also appeared to be an excessive quantity of clinical information provided which was probably unnecessary.  We wondered of the actor was also going to be the assessor and if so how feasible this is? If not should there be an additional set of instructions/ questions for the assessor. | I have reduced the amount of clinical info in the case study 1 |
|  | We don’t expect the HCW to know the questions but do expect them to ask the questions. The questions are in the hospital penA de-label guidelines. I think it is ok to have the questions on the sheet. |
|  | Now added |
|  | Re clinical info- it sets the scene to make it more realistic (based on a real case study). I have now made it more succinct. |
|  | Yes, the actor is the assessor. I think that is possible to deliver and makes the assessment more deliverable. Otherwise, the assessor would need to find a third person to do the assessment, which may be tricky. |
| Presentation – not reviewed in detail.  People commented on the busy nature of slides, and the quantity of the content. They also noted a large quantity of acronyms and wondered if a glossary of terms could be helpful.  Again has it been piloted on the target audience?  We felt that we should emphasise the need to not overestimate the ability of foundation doctors to have a working knowledge of the range of drug allergies.  The following comment about the slides was also sent to me after the session: The links between slides 11-13 need to be clarified (again heaps of info here in these slides). The link between the questions, Devchand’s tool and the proforma on slide 13 is unclear. Would the simulation use the proforma on slide 13 (does any trust/organisation)? In practice the questions on slide 11 (and which were used I think in the case study) seem too brief, whereas the proforma seems so detailed that most patients won’t be able to answer. | I don’t think I can reduce the content on the slides. This is the bare minimum that needs to be communicated to learners. I fear that if I remove content then I won’t convey key learning items.  Not yet piloted, but will be piloted.  The link will be given verbally. The slide set is pre-recorded and will describe the link between them i.e. take a penA focused history and use that history, with the decision support tool, to determine future risk of penA.  Yes, simulation / role play will use the slide 13 tool  The questions and the proforma come from eth same published manuscript and are designed to be used together. We’ve assessed >200 patients at RCHT using the combination and happy they work. |
| Overall comments:  The comment was made that an economic evaluation of the intervention could be useful. This could potentially be a very expensive intervention, every activity has an opportunity cost. Part of this evaluation/ consideration could include testing in a real-life population (for example one week of an acute medical take) to see how many patients this would actually apply to, i.e. how many patients would benefit from their penicillin allergy being de-labelled, versus how much time it would take (of assessor, participants) to achieve this?  Finally you also asked about other placed that this teaching could be taken. We thought that medical grand rounds perhaps with a case example of where this could have been useful could be a good place to target. | No HE evaluation planned due to low numbers of patients but we do plan to do one as part of a bigger study (iNAAN).  We have tested the process <https://pubmed.ncbi.nlm.nih.gov/37354996/> and happy with it |

**Appendix D PPIE review of PILs**

| **Reviewer name** | **Reviewers’ response / comments** | **Rebuttal / actions** |
| --- | --- | --- |
| 1 | I found them to be very interesting and informative, and very easy to understand. I believe if I had been given that information before, I would have been better informed about Penicillin allergies. | Nil to amend |
| 2 | A general comment would be that I am aware that making these documents into an actual leaflet will immediately make them more 'user friendly' | Actioned – that is the plan |
|  | PIL - 1  Although I can see difficulties with this leaflet I will admit to being at a loss to suggest how to improve it.  Sitting in the comfort of my own home I found this leaflet clear, extremely reassuring and interesting ( I had never thought about the differences between something causing an allergy or being simply a 'side effect') However, if taken to hospital and whilst feeling unwell and probably worried I don't think I'd take in all this information. The length of the document would overwhelm me, and the data would not necessarily register. In an ideal world this would easily be overcome by an health professionals explanation and reassurance - but would this happen? | Patient suggesting the information is provided verbally as well as written format which is proposed in the implementation intervention. |
|  | PIL2 & 3 When producing these 'leaflets' I think it needs to be restricted to one side of an A4 sheet, the minute you get to 'another page' it looses its impact and I believe most peoples interest. | PIL-3 is one side. PIL is 2 sided but can be potentially one sided – need to talk to publications |
|  | PIL2 Clear, reassuring , covers everything, I particularly like the clarity of the 'headlines' and the size of print. | Nil to amend |
|  | PIL3 Again clear and detailed enough. I like the fact that a card will be provided. | Removed reference to the card and suggested that they keep the leaflet with them. |
| 3 | Patient Information Leaflet (PIL) 1 - How easy is the leaflet to read and understand? Are there words or sentences in the leaflet that are unclear?  I’ve added comments and suggested changes to the actual documents. There are some instances where the language is a bit too clinical and could be misunderstood by patients, but overall I think the language is patient-friendly. It would be good for this to be produced by the graphic design team as an A5 leaflet in attractive colours. | Actioned. |
|  | Patient Information Leaflet (PIL) 1 - How reassuring is the leaflet for patients?  I think we could go further with this. The patient may perceive this process as going against previous medical advice which is a scary concept when you trust your medical teams, but the information is now conflicting with previous advice. | Address the fact that we are contradicting previous advice to avoid penicillin. This aligns with the WP2 patient interviews too – now addressed in PIL-1 |
|  | How persuasive is the leaflet? Would it encourage you to getting tested?  I didn’t find the leaflet persuasive, I found it informative. It didn’t have the word ‘choose’ in it anywhere, it was informing me of why we were going ahead with this process. I assumed this leaflet was being given out to people who had already agreed in principle to the process. If this is to be more persuasive, I would want to see the section on patient experience expanded much further, perhaps also some GPs they might recognise pictured in the leaflet with supportive quotes for the process. | A photo of a GP they might recognise will be tricky, unless it is a TV GP? Good idea, to discuss with local design and publications department.  Consider adding word ‘choose’. Not added but made it clear that PADL is “offered” not “done to”  Consider expanding the patient experience section- another quote added to the patient experience section. |
|  | How reassuring is the leaflet? Does it reassure you that testing is safe?  It gives lots of sensible argument for de-labelling, but I think I would like to see a bit more reassurance in it of what will happen if the patient has a reaction. The patient is taking a gamble that your new advice trumps that of the clinician in their history who labelled them in the first place and I think it would help to emphasis the ‘safe hands’ element of their care whilst going through this process. | Provide reassurance about what would happen if there was a reaction and that the patient is in safe hands. |
|  | Any other comments?  Trust and relationships might be stronger with GPs. Is this leaflet only for use in the acute setting? Perhaps we should be starting these conversations earlier in the primary care pathways. | Wider awareness raising – indeed. Nil to amend |
|  | PIL 2  How easy is the leaflet to read and understand? Are there words or sentences in the leaflet that are unclear?  No comments in relation to this, appears easy to understand  How reassuring is the leaflet for patients?  Yes  Do you think there needs to be any further information in here that isn’t?  A phone number to contact pharmacy as well as an email address for wider accessibility | Medicines Information number added to PIL. |
|  | PIL 2 - Any other comments?  Presumably this is the piece of paper we want patients to hang onto and many patients will be discharged with lots of pieces of paper in black and white text. Can this one be designed by RCHT Creative in a way which makes it stand out from the others and is more usable for the patient going forward? Perhaps they could also be given a little card to say they are NOT allergic to penicillin to remind them to reinforce this for future clinical contacts? | Redesign the leaflet so it is colourful and appealing – we are constrained by the ward printers (black and white).  The challenge with having cards is where to store them. |
|  | PIL 3 - How easy is the leaflet to read and understand? Are there words or sentences in the leaflet that are unclear?  No comments in relation to this, appears easy to understand.  Do you think there needs to be any further information in here that isn’t?  Contact details for questions post-discharge (eg PALS)  Any other comments?  As above, graphic design of these to make them more appealing and more likely to be retained post-discharge would be good. | I don’t think PALS is appropriate. We’ve said to contact their medical team and now added in an email and a telephone number. |
| 4 | How easy is the leaflet to read and understand? Are there words or sentences in the leaflet that are unclear? – **Yes, although see below, I have suggested some changes**  How reassuring is the leaflet for patients? – **The leaflet made sense to me, and you have covered the reasons why – personally, I would be reassured**  How persuasive is the leaflet? Would it encourage you to getting tested? – **Whilst I would be nervous, personally, yes, I would get tested**  How reassuring is the leaflet? Does it reassure you that testing is safe? – **The information provided is clear, but as the patient would be an in-patient, may be a little more reassurance around being closely monitored?** | Now made clearer in the leaflet that they are in safe hands. |
|  | **PIL1**  PAGE 1  Paragraph 1 – I have added in a comment in the second line (as highlighted)  Paragraph 2 -  highlighted “This included” – may I suggest:  **These include** instead?  I also added a comment after the word childhood, as highlighted  Paragraph 4 – highlighted **WITH** should read **WILL**.  I had to read the rest of the paragraph a couple of times before it made sense to me.  I think by changing the wording it may make more sense.  Suggestion: **Patients that report having had a mild side effect, or an intolerance, can have their allergy record removed without testing.** **These intolerances may include things like feeling sick or having diarrhoea.**  (if I have understood it correctly)  Paragrah 5 – have added in a couple of commas, as highlighted  Paragraph 6 – have added in commas, as highlighted  PAGE 2  Paragraph 3 – **THRUST** should read **THRUSH**, as highlighted | All actioned |
|  | **PIL 2**  How easy is the leaflet to read and understand? Are there words or sentences in the leaflet that are unclear? – **Yes easy.  No – I found it very clear**  How reassuring is the leaflet for patients? – **personally, I would feel reassured**  Do you think there needs to be any further information in here that isn’t?  Any other comments?  **PIL2**  Paragraph 4 – you appear to start the paragraph in the past tense, therefore should **DEVELOPS** be **DEVELOPED**? (highlighted in pink)  **KNOWN** should read **KNOW** – as highlighted in pink | All actioned |
|  | **PIL 3**  How easy is the leaflet to read and understand? Are there words or sentences in the leaflet that are unclear? – **Easy to read – nothing is unclear to me**  Do you think there needs to be any further information in here that isn’t? – **Nothing I can think of**  Any other comments?  **as below**  PIL3  No change – although may I suggest the following should be in red?  It’s just a thought  ***YOU*** ***ARE ALLERGIC TO PENICILLIN***  *You should continue to avoid taking antibiotics which contain penicillin.* | Not actioned, as colour printing is not available in ward and departmental areas. |
|  | The only other suggestion I would make to all documents is to justify them. They look a little neater when all the paragraphs end at the same place |  |

**Appendix E**

| Implementation outcome organised by Consolidated Framework for Implementation Research (CFIR) Outcomes Addendum^34^ group | | |
| --- | --- | --- |
| **Adoption** | **Method (how measured)** | **Measurement** |
| Hospital organisational level support for PADL | Medicines Practice Committee approval of hospital PADL guidelines and support for non-allergist delivery of PADL within the defined governance framework. | PADL guidelines published on the hospital’s intranet.  Minutes from MPC evidencing the committee’s support for PADL. |
| Senior doctor acceptance of PADL; (A) agreeing to their patients being tested and (B) testing of their own patients | PADL champion attendance at specialty meetings to discuss PADL with senior specialty clinicians to gauge support for PADL. | A log of medical and surgical specialties that accept the invitation to discuss PADL in their specialties with the PADL champion.  Count of number of medical and surgical specialties departments agreeing to (A) authorise PADL for their patients (B) undertake PADL for their patients. |
| Nurses willing to participate in the required post DOC observations. | PADL champion attendance at specialty meetings with nurse representation and Heads of Nursing meetings. AMS team to note any nurse refusal to deliver post DOC observations during implementation. | A record of meetings attended by the PADL champion, and a record made of the sense of willingness to engage with PADL determined through positive and negative feedback from the members of those groups. A log made of senior nurse meeting awareness raising interactions and a record made of the sense of willingness to engage with post -DOC observations. Note any nurse refusal or concerns to undertake post DOC observations during implementation at ward level |
| **Implementation** | **Method (how measured)** | **Measurement** |
| PenA focused histories are being taken during inpatient stays by ward pharmacy teams as part of medication reconciliation or medical teams as part of inpatient care. | For all patients with a penA record, the number with a penA focused history documented as a ‘PADL note’ in EPMA and numbers without prior to discharge from hospital. | Percentage of penA patients with a ‘PADL’ note by the time of discharge, presented by month of discharge from hospital. |
|  | For all patients with a penA label A who received antibiotics during their inpatient stay, the number with a penA focused history documented as a ’PADL’ note and numbers without prior to discharge. | Percentage of penA patients on any antibiotic with a ‘PADL’ note by the time of discharge, presented by month of discharge from hospital. |
|  | For all patients with a penA label, name and grade of staff who added the penA history as a ‘PADL note’. | Grade of staff presented as a percentage of all staff adding ‘PADL’ notes. |
| Determine who is risk assessing penA focused histories within EPMA notes and making the decision to de-label (either DOC or DDL). | For all patients with a penA history, who risk assessed the patient. | Staff groups risk assessing PADL notes as a percentage of all PADL notes assessed. |
|  | Identify which specialties patients are under when PADL’d. | Collate the name of the specialty the patient was under when PADL’d |
|  | Identify which clinical teams are initiating PADL and de-labelling themselves. | Which Specialty doctors/pharmacists delivering PADL |
|  | Of those de-labelled (DDL or DOC) a review of the EPMA records and medical notes to determine who made the decision to PADL. | Staff groups de-labelling patients as a percentage of all de-labelled patients. |
| Determine missed opportunity to de-label through:    (A) Patients discharged with a penA record, received an antibiotic and without a penA ‘EPMA’ note. | Identify patients discharged with a penA record and received an antibiotic with a ‘PADL’ note without evidence of having been risk assessed. | The percentage of patients discharged with a penA record who received antibiotic during their inpatient stay but without a PADL EPMA note. |
| (B) A review of those that have a penA focused history documented in EPMA but which have not been risk assessed. | Identify patients discharged with a penA record and received an antibiotic with a ‘PADL’ note without evidence of having been risk assessed. | For patients with a penA record and received antibiotics during their stay, the percentage of ‘PADL’ notes not risk assessed prior to discharge. |
| (C) A review those that have been identified as low risk, but not de-labelled; to identify reasons for not being de-labelled. | Identify patients discharged with a penA record and received an antibiotic with ‘PADL’ notes that have been risk assessed as low risk but hasn’t been de-labelled. | Proportion of low-risk patients not de-labelled. |
| The following PADL steps are undertaken for each patient de-labelled by either DDL or DOC:   - Appropriate penA history taken. - Appropriate penA risk assessment, category assigned. - DOC prescribed appropriately. - post DOC observation completed. - patients counselled and consented. - patient given PIL. - EPMA records amended. - Evidence of GP communication. | Review of patient written and electronic medical notes and electronic prescribing records of de-labelled patients. | Each step presented as percentage compliance with that step. |
| Uptake of training | Numbers of HCWS (grade, specialty at time of sign off) completing part 1 and part 2 of PADL training within first three months of launch. | Numbers of HCWs by staff group completing part 1 training and presented as a percentage, where able.  Numbers of HCWs completing part 2. |
|  | Ongoing log of training against a list of ward pharmacy technicians and pharmacists and foundation years doctors. And a log of other staff who have completed training. | Numbers of HCWs who have complete part 1 and part over the implementation period (6 months). |
| Compliance with training requirements | Whether the HCW risk assessing penA records and HCW de-labelling patients have undertaken requisite training and cross referenced against a register of HCWs who have completed training. | Percentage of HCWs assessing and percentage of HCWs de-labelling having undertaken the part 1 and part 2 training. |
| Effectiveness of nursing communication channels | Survey emailed to ward nurses to determine whether the message about PADL had reached the ward nurses. Have the ward sisters communicated the benefits and risks and their support for PADL? Have they seen the A4 document? Do they have concerns with PADL and delivery of DOC observations? | From the nurse interviews and the emailed survey, percentage of nurse respondents that had received communications about PADL from senior nurse colleagues. |
|  | One-to-one nurse interviews (see below) and nurse survey sent via email to adult inpatient ward sisters for completion and cascade to staff nurses |  |
| Knowledge of PADL champions | Do doctors and pharmacists know who the PADL champions are, and are they accessible? One-to-one interviews and email survey. | Percentage of survey respondents aware of who the PADL champions are and percentage reporting them to be accessible. |
| Acceptability, appropriateness and feasibility of PADL | How measured | Measurement |
| Acceptability: The extent to which PADL is perceived as “agreeable, palatable, or satisfactory.”  Appropriateness: The perceived fit, relevance, or compatibility of PADL delivered in the inpatient setting.  Feasibility: The extent to which PADL can be successfully used or carried out within the inpatient setting. | We will use the RE-AIM QuEST framework for programme evaluation to identify and explore real-time implementation barriers using semi structured intervews.^35^  During the first 12-24 weeks of implementation, ten to fifteen patients meeting the de-labelling criteria will be invited to interview to explore their experiences, their beliefs and their concerns about the penicillin allergy de-labelling process using semi-structured interviews. | To Explore the patients experiences of PADL; the barriers and enablers to accepting PADL and ways to improve the process. |
|  | At week 24 post implementation ten to fifteen healthcare workers (nurses, doctors and pharmacists) will be interviewed to gain insight into their experiences of the penicillin allergy de-labelling process. | To determine whether the intervention has changed healthcare worker behaviour towards/around assessment of penA/PADL.  To determine the wider clinical team barriers and enablers to their engagement with PADL and explore senior doctor acceptance of PADL and their willingness to consider PADL for their patients. |
| PADL (innovation) outcome measures | How measured | Measurement |
| Recipient (patient) outcomes:  Number of patients de-labelled by DDL and DOC | Each month, the data will be reviewed to determine how many patients were risk assessed by HCWs and the number successfully de-labelled by either DDL or DOC. | By month of discharge from hospital, the number of patients de-labelled by DDL and DOC and presented as percentage of the following denominators; of those risk assessed, of those with a penicillin allergy record and prescribed antibiotics, of those with a PADL note and of those admitted with a penicillin allergy label. |
| Determine whether PADL increases penicillin antibiotic use in adult medical and surgical specialties and reduces non-penicillin antibiotic use. | Antibiotic consumption data for each patient spell will be extracted from the EPMA system. For patients de-labelled, the de-label date will separate the antibiotic use before and after de-label. The antibiotics will be grouped by the UK version of WHO AWaRe category and converted to DDDs. | Antibiotic DDDs by AWARE category will be compared between the following groups;  First comparison:   - PenA Patients not de-labelled during inpatient spell - PenA Patients de-labelled during inpatient spell   Second comparison   - PenA patients not de-labelled during inpatient spell plus antibiotics pre-PADL date for those de-labelled - Antibiotics post PADL date in those de-labelled.   Third comparison:  Any readmission spells within 90 days of discharge   - PenA patients not de-labelled - PenA patients de-labelled. |
| Determine the PADL-related harm. | Notes review at 5 days for patients who have been risk assessed and de-labelled and those risk assessed and not de-labelled to determine whether there has been any antibiotic related harm due to antibiotic exposure (either penicillin antibiotic exposure or non-penicillin antibiotic exposure). | Proportion of patients’ risk assessed and not de-labelled, or risk assessed and de-labelled but not exposed to a penicillin, who experience antibiotic-associated harm.  Proportion of patients’ risk assessed, de-labelled and exposed to a penicillin antibiotic who experience penicillin-associated harm. |
| Numbers of PADL patients with updated GP records at 90 days post discharge from hospital. | Patients de-labelled, either by DDL or DOC, will be first checked to see whether they had a penA in their GP records prior to admission (collected as part of the service improvement data collection). Those without a GP record of penA prior to admission will be excluded. The proportion of those remaining with a retained PenA record will be determined. | Proportion patients de-labelled who retained their GP penA record three months post discharge. |
| Explore the impact of PADL on patients through qualitative research. | (As described above) during the first 12-24 weeks of implementation, ten to fifteen patients meeting the de-labelling criteria seven days post discharge will be invited to interview to explore their experiences, their beliefs and their concerns about the penicillin allergy de-labelling. |  |
| Deliverers (healthcare workers)  Explore doctor, pharmacist, MOPT and nurse views on PADL to determine their beliefs on whether PADL improves the patient experience and improves population health and what impact it might have on cost and the impact of PADL on the HCWs experiences at work (to determine whether it negatively impacts work-life). | (As above) At week 24 post implementation ten to fifteen healthcare workers (nurses, doctors and pharmacists) will be interviewed to gain insight into their experiences of the penicillin allergy de-labelling process. |  |

Table A2 Intervention implementation outcome measures for PADL intervention.
