# Supplementary material for: Description of the combined evidence-based, theory-based and person-based approaches used to develop a behavioural intervention package to support non-allergist healthcare workers to remove incorrect penicillin allergy records from medical and surgical adult inpatients in a UK hospital
Source: BMJ Open. 2025 Jul 30;15(7):e096452. doi: 10.1136/bmjopen-2024-096452 (PMC12315033; doi:10.1136/bmjopen-2024-096452)
Supplement: Supplementary file 2 [file bmjopen-2024-096452.reviewer_comments.pdf]

PEER REVIEW HISTORY

BMJ Open publishes all reviews undertaken for accepted manuscripts. Reviewers are asked to complete a checklist review form and are provided with free text boxes to elaborate on their assessment. These free text comments are reproduced below.

ARTICLE DETAILS

Title (Provisional)

Description of the combined evidence based, theory-based and person-based approaches used to develop a behavioural intervention package to support non-allergist healthcare workers to remove incorrect penicillin allergy records from medical and surgical adult inpatients in a UK hospital.

Authors

Powell, Neil; UPTON, Mathew; Kent, Bridie; Sandoe, Jonathan; Tonkin-Crine, Sarah

---

VERSION 1 - REVIEW

**Reviewer**	**1**
**Name**	**Li, Philip**
**Affiliation**	**Hong Kong University**
**Date**	**21-Dec-2024**
**COI**	**None**

Thank you for the opportunity to review this manuscript. I commend the authors for their effort in writing up this manuscript and tackling the niche issue of how to encourage staff to setup delabelling initiatives.

Despite the very comprehensive explanation on all the various interview techniques used, this work is essentially a descriptive/"qualitative" summary of a series of small Microscoft Team "workshops" among a small group (8) of people from 1 specific location (and single hospital) in the UK. It therefore lacks external validity and may not be as relevant for the wider audience. The majority of the paper seems to focus on the methodology and the process, while the actual results are descriptive and comparatively underwhelming. Especially with the seemingly lack (or unclear) action points or suggestions, overall this study seems less beneficial to a wider audience

Furthermore, most of the tables / figures are not describing original data, and can be supplementary. Overall, if only original data/results were condensed, this study may be more suitable for a brief report/research letter.

**Reviewer**	**2**

**Name**	**Blumenthal, Kimberly G**
**Affiliation**	**Massachusetts General Hospital**
**Date**	**03-Jan-2025**
**COI**	**None**

This article describes a comprehensive implementation package to reduce the negative impact of penicillin allergy mislabels.

My comments for improvement are not extensive but related to:

1. Choice of frameworks - Could the choice of framework (TDF, CFIR etc) and the step(s) it is used in, purpose/choice, and reference be included in a summary table or methods.

2. Quantitative outcomes - can the outcomes that are quantitative be summarized to include how they will be presented and compared? Are any quantitative analyses powered to determine a difference between periods? Is there a statistical analysis plan for any quantitative outcomes? What is the methodologic approach?

3. Outcomes in general - are any of the outcomes in RE-AIM primary outcomes? Which are the main outcomes and like in #2 above, are any being powered and/or have a prespecified analytic approach?

4. Discussion - are there any prior implementation science studies in this space to compare to?

**Reviewer**	**3**
**Name**	**Trubiano, Jason**
**Affiliation**	**Austin Health, Infectious Diseases**
**Date**	**18-Jan-2025**
**COI**	**None**

Thank you for the opportunity to review this well written manuscript. This is a well written manuscript in a difficult area of health services research addressing a key AMS target. I have very few suggesitons for improvement.I would however see if some of the methods could be moved to the supplementary and summarised more in text. It can be quite dense and difficult to read through the text, but understanding its a difficult concept to simply in a small number of words. Some minor suggestions for improvement are included below:

MINOR

Line 60 – It would be good to confirm this section was following healthcare worker interview. Can "" to support the findings also be included in text. In addition the n % that reported each theme/statement.

Line 23 – Is it possible to do similar here with providing some quantitative support to “most”. Understandable as this is primarily an implementation and qualitative project, but it might help the non qualitative reader understand the significance.

Line 42 – Where the research from the hospital where the program/implementation was deployed?

VERSION 1 - AUTHOR RESPONSE

Reviewer: 1<br>Dr. Philip Li, Hong Kong University	
Thank you for the opportunity to review this manuscript. I commend the authors for their effort in writing up this manuscript and tackling the niche issue of how to encourage staff to setup delabelling initiatives.	We are not exploring how to encourage the setting up of penicillin allergy de-labelling initiative. The manuscript describes the development of an implementation intervention designed to encourage and support non-allergists to de-label.
Despite the very comprehensive explanation on all the various interview techniques used, this work is essentially a descriptive/"qualitative" summary of a series of small Microsoft Team "workshops" among a small group (8) of people from 1 specific location (and single hospital) in the UK. It therefore lacks external validity and may not be as relevant for the wider audience.<br><br>The majority of the paper seems to focus on the methodology and the process, while the actual results are descriptive and comparatively underwhelming. Especially with the seemingly lack (or unclear) action points or suggestions, overall, this study seems less beneficial to a wider audience	As per the assistant editor above, we believe there has been a misunderstanding of what this work is describing. This work is a comprehensive explanation of an integrated approach to developing an implementation intervention using behaviour change theory, evidence from qualitative studies and the Person-Based Approach.<br><br>For clarification, the Microsoft Teams workshops included 9 healthcare workers external to the study hospital, 10 healthcare workers from the study hospital and 8 external medical education experts; a total of 27 participants of which 17 (63%) were external to the study hospital.<br><br>The study presents the methods for developing an intervention which are generalisable to other NHS and non-UK settings and provides examples of intervention components that may be applicable for our PADL intervention and other healthcare settings. The output in table 3 and figure 2 are a proposed actions informed by the study methods.
Furthermore, most of the tables / figures are not describing original data, and can be supplementary. Overall, if only original data/results were condensed, this study may be more suitable for a brief report/research letter.	The previous published work is briefly described and the main findings from the previous work brought together to inform the development of this implementation intervention, which is well accepted methodology. The data in the figures and tables are all describing original data.

Reviewer: 2<br>Dr. Kimberly G Blumenthal, Massachusetts General Hospital	
Comments to the Author:<br>This article describes a comprehensive implementation package to reduce the negative impact of penicillin allergy mislabels.	
My comments for improvement are not extensive but related to:<br>1. Choice of frameworks - Could the choice of framework (TDF, CFIR etc) and the step(s) it is used in, purpose/choice, and reference be included in a summary table or methods.	Thank you, we have made the following changes: Under methods section "Stage 2. Implementation intervention planning, design & development" the COM-B and TDF are now referenced. The TDF and BCW/COM-B are referred to and referenced in the method sections that utilise these frameworks. The CFIR is utilised in stage 4 and already referenced in the methods with the results presented in table 4. Table 1 shows the implementation intervention development steps. To further improve clarity, we have added a column to table 1 that shows the stages in which the TDF, BCW or CFIR were used.
2. Quantitative outcomes - can the outcomes that are quantitative be summarized to include how they will be presented and compared?<br><br>Are any quantitative analyses powered to determine a difference between periods?<br><br>Is there a statistical analysis plan for any quantitative outcomes?<br><br>What is the methodologic approach?	This pertains to 'PADL (innovation) outcome measures in table 4. We agree, how the quantitative outcome measures will be measured and presented is important and will form part of the evaluation of how successful the implementation intervention is. We have used the CFIR to propose outcome measures for both the implementation strategy and the intervention (PADL). Although we feel it is beyond the scope of this paper to report details of the statistical analysis (and the next paper will report the intervention delivery and report the quantitative and qualitative outcomes with the statistical analysis methods used to evaluate those) we have alluded to the likely need for a quasi-experimental design. Funding and time constraints preclude an RCT so we are left with quasi-experimental designs.
3. Outcomes in general - are any of the outcomes in RE-AIM primary outcomes? Which are the main outcomes and like in #2 above, are any being powered and/or have a prespecified analytic approach?	The primary quantitative outcome measure is non-Access antibiotic use comparing those that are de-labelled with those that are not. The statistical analysis will be a logistic regression with potential confounders and de-labelled status.

	Assuming non-Access (WHO antibiotic classifications) antibiotic exposure in those labelled penA decreased from 50% pre-de-label to 30% if de-labelled, based on some previous work in our hospital, and with 80% power, and we assume that one third of penA patients are de-labelled, a sample size of 231 (77 de-labelled + 154 not de-labelled) would be required to detect a statistical difference. From previous work undertaken in the study hospital we expect to exceed these numbers in a 6 month period.
4. Discussion - are there any prior implementation science studies in this space to compare to?	The ALABAMA study reported their penicillin allergy de-labelling implementation development process and is referenced in the discussion (ref 24). However, that was a primary care intervention. The only other penicillin allergy de-labelling implementation science study is your own 2017 study (ref 41). We have compared our study to both of these studies as well as other antibiotic stewardship implementation science studies outside of penA de-labelling (refs 23 & 25).<br><br>If there are others that we are unaware of then please do highlight these to us for consideration.
Reviewer: 3<br>Dr. Jason Trubiano, Austin Health<br>Comments to the Author:	
Thank you for the opportunity to review this well written manuscript. This is a well written manuscript in a difficult area of health services research addressing a key AMS target. I have very few suggestions for improvement.	
I would however see if some of the methods could be moved to the supplementary and summarised more in text. It can be quite dense and difficult to read through the text, but understanding its a difficult concept to simply in a small number of words. Some minor suggestions for improvement are included below:	We agree the text is dense. Communicating the multiple methods and outcomes in this study is a challenge and reducing the word count further would make it more difficult for readers to replicate the methods and to understand our key findings without referring to the appendix. As such we believe that moving some of the methods sections to the appendix will detract from the manuscript.
MINOR<br>Line 60 – It would be good to confirm this section was following healthcare worker interview. Can “” to support the findings also	(line 60 on page 5). We have now added ‘interviews’ to the subheading to make it clear that these findings are following the interviews. This section provides a summary of the findings in reference 31. In reference

be included in text. In addition, the n % that reported each theme/statement.	31 we report it as 'most' and have not provided numbers of respondents who expressed those opinions. As such we are not able to provide numbers in here either. It would not be usual practice to estimate the number of people with a particular view in purposively sampled interview population.
Line 23 – Is it possible to do similar here with providing some quantitative support to "most". Understandable as this is primarily an implementation and qualitative project, but it might help the non-qualitative reader understand the significance.	(Page 6, line 23). Understood, but as above, in this section we summarise reference 32 which does not report the data quantitively.
Line 42 – Where the research from the hospital where the program/implementation was deployed?	(page 6 line 42). We have included a few words to make it clear that the pharmacist works at the hospital and that the health psychologist and the medical microbiologist do not work at the study hospital.

VERSION 2 - REVIEW

Reviewer 3

Name Trubiano, Jason

Affiliation Austin Health, Infectious Diseases

Date 13-Jun-2025

COI

All requested addressed
